# Facilitating Expedited Drug Approval—the Past, Present, and Future of Clinical Trials for Pediatric Rheumatic Disease

**DOI:** 10.1002/acr.80028

**Published:** 2026-04-07

**Authors:** Pamela F. Weiss, Hermine I. Brunner

**Affiliations:** ^1^ Department of Pediatrics and Center for Clinical Epidemiology and Biostatistics, Perelman School of Medicine University of Pennsylvania, Division of Rheumatology and Clinical Futures, Children's Hospital of Philadelphia Philadelphia Pennsylvania; ^2^ Department of Pediatrics University of Cincinnati, Cincinnati Children's Hospital Medical Center Cincinnati Ohio

## Introduction

Over the past five decades, pediatric rheumatology has undergone a significant transformation in the design, conduct, and impact of clinical trials, evolving from small, largely exploratory studies to sophisticated, multinational research programs that have reshaped the standards of care for children with rheumatic diseases. Between 1975 and 1995, the field was characterized by single‐center trials focused on nonsteroidal anti‐inflammatory drugs (NSAIDs), glucocorticoids, and early conventional disease‐modifying antirheumatic drugs (cDMARDs), constrained by small sample sizes, heterogeneous outcome measures, and the absence of standardized disease definitions. The period from 1996 to 2010 was a critical phase of consolidation and framework building, characterized by the introduction of unified disease classification systems, such as the International League of Associations for Rheumatology juvenile idiopathic arthritis (JIA) criteria,^1^ the development of consensus outcome measures, and the expansion of multicenter trials evaluating methotrexate and the first biologic therapies. These advances were catalyzed by the formation of collaborative research networks and registries that enabled enrollment at scale and fostered methodologic rigor. From 2011 to the present, the field of pediatric rheumatology has entered a modern era defined by rapid growth in biologic and targeted small‐molecule trials, the adoption of innovative trial designs, and the systematic use of registries to assess long‐term safety and effectiveness. Together with strengthened regulatory incentives and internationally coordinated consortia, these advances have fundamentally altered the clinical trial landscape, accelerating therapeutic innovation while improving the quality and relevance of evidence generated for children with rheumatic diseases.

## Regulatory, ethical, and logistical landscape

Over the past 50 years, the regulatory landscape for pediatric clinical trials has shifted from a largely permissive environment to one that emphasizes safety, efficacy, and ethical oversight. In the early 1970s, children were often excluded from clinical research, and most medications were prescribed off label without robust pediatric data, exposing young patients with rheumatic diseases to unknown risks. Growing recognition of this problem, along with activism by professional organizations, pediatric research networks, families, and stakeholder organizations, led to pivotal legislative and regulatory changes, most notably the Best Pharmaceuticals for Children Act (BPCA, 2002)^2^ and the Pediatric Research Equity Act (PREA, 2003).^3^ BPCA encouraged pharmaceutical companies to study their drugs in children by offering a six‐month extension of marketing exclusivity. PREA went further by mandating that pharmaceutical companies conduct pediatric studies for new drugs and biologics if they are likely to be used in children or if there is a pediatric correlate of a non‐orphan disease occurring in adults. PREA also mandated that the pediatric study plans be submitted before the adult disease counterpart phase 3 trial commences.

In 2012, the US Food and Drug Administration (FDA) Safety and Innovation Act made PREA and BPCA permanent, which has been pivotal for advancing pediatric drug development. In 2012, as part of the FDA Safety and Innovation Act, Congress established the Pediatric Rare Disease Priority Waiver Program. When a company receives FDA approval for a therapy for a pediatric rare disease that qualifies under this program, it is awarded a priority review voucher. This voucher can then be used to expedite the FDA review process for another product that would otherwise not qualify, reducing the standard 10 to 12 months to 6 months. The large financial incentive from the program comes from the fact that the voucher can also be sold or transferred to another company. In 2024, the Center for Drug Evaluation and Research (CDER) E11A Pediatric Extrapolation Guidance for Industry provided recommendations for how to present extrapolation plans in regulatory submissions and how to engage with the FDA during drug development.

Collectively, these laws have facilitated the labeling of numerous drugs for children, including those with JIA and pediatric systemic lupus erythematosus (pSLE). Internationally, guidelines from the European Medicines Agency and the International Council for Harmonisation (ICH) have promoted age‐appropriate formulations, dosing, and trial design in a similar manner. Over time, regulatory agencies have increasingly emphasized ethical protections, including obtaining assent from minors and parental consent, while also minimizing trial‐related burdens and fostering innovation in pediatric trial methodology. Today, the landscape reflects a delicate balance: ensuring children benefit from advances in medicine while protecting them from unnecessary harm, a paradigm shift that continues to shape the design and conduct of pediatric research worldwide. Progress, however, is still needed as off‐label use of medication is still common, accounting for 40% of drugs prescribed in the pediatric population.^4^

## Pediatric rheumatology research networks and clinical trials

Several stakeholder organizations have played pivotal and complementary roles in advancing the design, conduct, and global impact of clinical trials in pediatric rheumatology over the past 50 years. The Pediatric Rheumatology Collaborative Study Group (PRCSG), established in 1973, was the first formal pediatric rheumatology research network, laying the groundwork for the development of modern clinical trials in the field. PRCSG pioneered multinational pediatric trials by coordinating studies conducted in the United States and the former Union of Soviet Socialist Republics (USSR). Through rigorously designed, placebo‐controlled trials, PRCSG evaluated cDMARDs, including D‐penicillamine,^5^ hydroxychloroquine,^5^ oral gold,^6^, ^7^ NSAIDs,^8^, ^9^ and methotrexate,^10^ for the treatment of what was then known as juvenile rheumatoid arthritis. These trials demonstrated a lack of efficacy for several of the agents while establishing methotrexate and NSAIDs as effective therapies, thereby defining the therapeutic foundation for JIA. PRCSG subsequently led the first biologic clinical trials in pediatric rheumatology, including the inaugural tumor necrosis factor inhibitor trial of etanercept in JIA,^11^ which ultimately supported FDA approval for it in polyarticular juvenile rheumatoid arthritis in 1999. Over five decades, PRCSG has maintained a critical role in trial design, execution, and dissemination through sustained partnerships with industry sponsors.

Building on this early momentum, the Paediatric Rheumatology International Trials Organisation (PRINTO) was founded in 1996 to address the growing need for large‐scale, multinational pediatric rheumatology trials. PRINTO created the infrastructure necessary to conduct harmonized, rigorously designed clinical trials across diverse geographic and regulatory environments. By enabling the enrollment of sufficiently powered cohorts for rare pediatric rheumatic diseases, PRINTO has played a crucial role in advancing therapeutic development, validating outcome measures, and securing regulatory approvals in Europe and globally. Its emphasis on standardized protocols, centralized data coordination, and international collaboration has made PRINTO a cornerstone of global pediatric rheumatology clinical trials and research.

In 2000, the Childhood Arthritis and Rheumatology Research Alliance (CARRA) was established to advance collaborative clinical research across North America, with a particular focus on generating real‐world evidence. A major milestone was the launch of the CARRA Registry in 2009, the first national registry for pediatric rheumatic diseases, alongside the development of robust trial infrastructure, including centralized site management, standardized contracting, site training, and a dedicated research coordinator network. Today, investigators from more than 120 institutions contribute data to the registry, which has supported landmark interventional and comparative effectiveness studies, including the Trial of Early Aggressive Therapy in Polyarticular JIA (TREAT‐JIA).^12^ In 2015, the registry was upgraded to be 21 Code of Federal Regulations (CFR) Part 11–compliant, enabling its use for global postmarketing safety surveillance and the assessment of rare but critical long‐term outcomes, such as malignancy.

## Clinical trial design

The earliest trials of conventional synthetic DMARDs in juvenile rheumatoid arthritis, such as D‐penicillamine,^5^ hydroxychloroquine,^5^ oral gold,^7^ and methotrexate,^10^ relied on the traditional randomized, placebo‐controlled design. Once effective treatments, most notably methotrexate, were established, the continued use of placebo controls in children, particularly those with a high burden of disease, became increasingly difficult to justify ethically. This shift necessitated the development of alternative trial methodologies, including randomized withdrawal designs, open‐label pediatric pharmacokinetic studies, and approaches to efficacy extrapolation.

### Randomized withdrawal design

**Figure 1:**
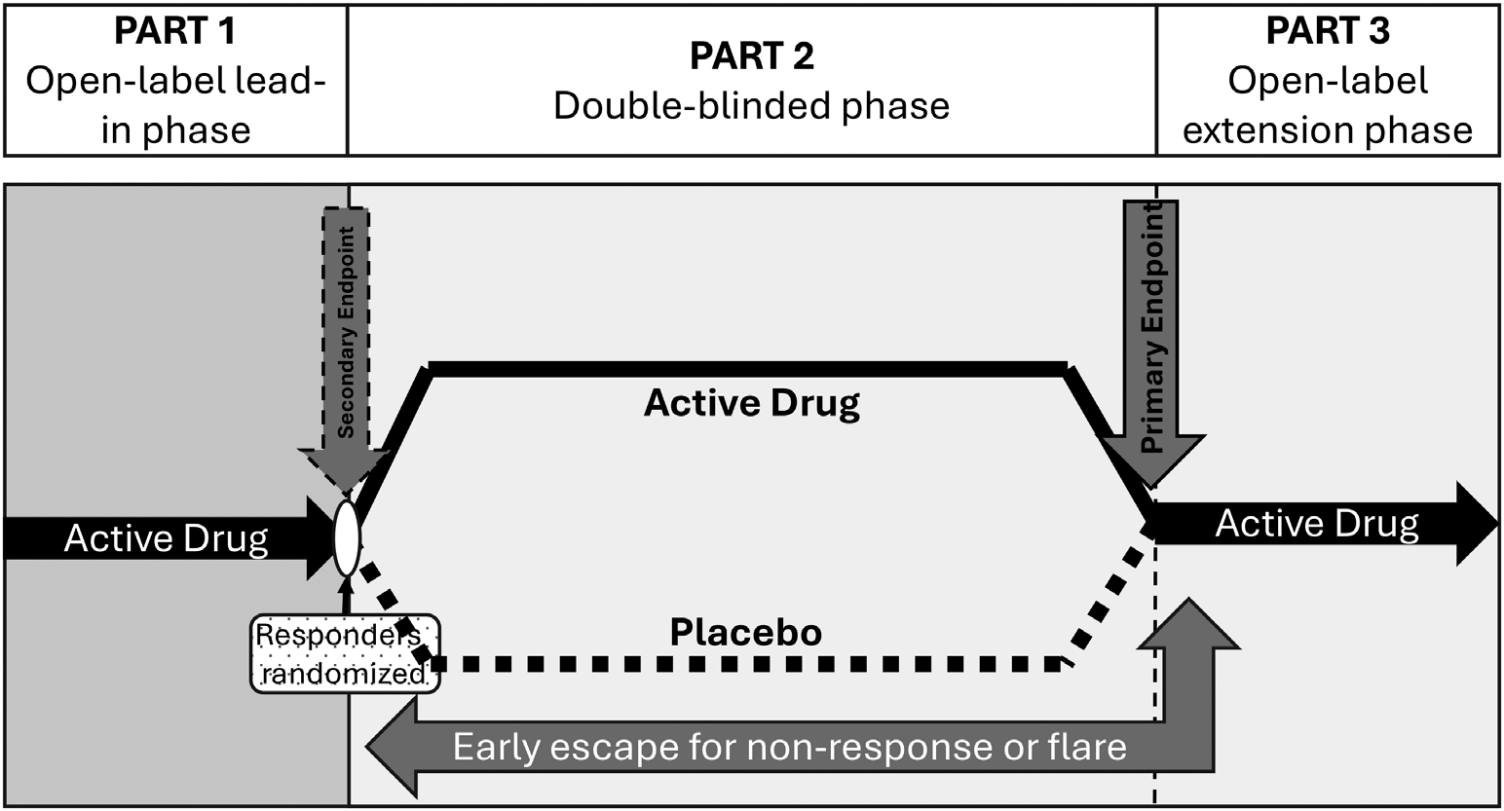
Randomized withdrawal design. Figure adapted from “Pediatric Rheumatology Collaborative Study Group ‐ over four decades of pivotal clinical drug research in pediatric rheumatology.”^46^

The first biologic DMARD studied in JIA, etanercept, was evaluated using the novel randomized withdrawal trial design (RWD)^11^ (Figure 1). In this framework, all enrolled children receive open‐label treatment with the experimental therapy during the initial phase of the trial. Participants who achieve a predefined clinical response, typically a JIA American College of Rheumatology (ACR) 30 response,^13^ were subsequently randomized in phase 2 to either continue the investigational therapy or transition to a placebo. During phase 2, participants were then monitored for a protocol‐defined disease flare or time to flare. Children who experienced a mild to moderate flare had the option to restart the active investigational drug in open‐label extension (phase 3). The RWD design facilitates efficient assessment of drug efficacy while minimizing placebo exposure and enabling individualized treatment duration. Importantly, this design has greater acceptability among clinicians and families because all participants receive the active experimental therapy immediately after enrolling into the study, which generally coincides with active disease and pain. Further, time on placebo is minimized, and there is an option to resume the drug in phase 3. However, the RWDs introduce selection bias, as only responders advance to randomization in phase 2. Additionally, the principal outcome, time to disease flare, does not directly compare response rates. This design is not suitable for therapies with a prolonged effect or when abrupt discontinuation of the drug raises safety concerns. Despite these limitations, most biologic trials in juvenile arthritis conducted between 1999 and 2020 employed an RWD (Table 1).

**Table 1:** Drug approvals and trial design supporting the approval from 1999 to present*

Drug	Year of FDA pediatric approval	Indication(s)	Trial design supporting pediatric approval
Etanercept	1999	Polyarticular JRA	Randomized withdrawal
Adalimumab	2008	Polyarticular JRA	RCT + open‐label extension
Abatacept	2008	Moderate–severe pJIA	Randomized withdrawal
Tocilizumab	2011	Systemic JIA	Randomized withdrawal
	2013	pJIA	Randomized withdrawal
Canakinumab	2013	Systemic JIA	Randomized withdrawal
Golimumab	2020	pJIA; juvenile psoriatic arthritis	Extrapolation from adult RA/PsA + open‐label pediatric PK/safety study
Tofacitinib	2020	Polyarticular‐course JIA	Randomized withdrawal
Secukinumab	2021	Juvenile psoriatic arthritis; ERA	RCT (randomized withdrawal)
Ustekinumab	2022	Juvenile psoriatic arthritis	Extrapolation from adult PsA + open‐label pediatric study
Sarilumab	2024	pJIA	Extrapolation + pediatric open‐label PK/safety study
Guselkumab	2025	Juvenile psoriatic arthritis	Extrapolation from adult PsA + pediatric PK/safety study

* ERA, enthesitis‐related arthritis; FDA, US Food and Drug Administration; JIA, juvenile idiopathic arthritis; JRA, juvenile RA; pJIA, polyarticular JIA; PK, pharmacokinetics study; PsA, psoriatic arthritis; RA, rheumatoid arthritis; RCT, randomized controlled trial.

### Extrapolation studies

In parallel, regulatory pathways evolved to further accelerate pediatric drug development. In 1994, the FDA first proposed extrapolating efficacy data from adult trials when disease pathophysiology and treatment response were sufficiently similar in adult and pediatric populations. Under this paradigm, pediatric development could rely on pharmacokinetic and safety data from relatively small open‐label studies in children. In 2019, an FDA/Centers for Excellence in Regulatory Science and Innovation workshop addressed strategies to accelerate drug development for children with polyarticular JIA.^14^ After this workshop, the FDA determined that PREA requirements for polyarticular JIA could be satisfied through pharmacokinetic studies supporting extrapolation of efficacy from adult rheumatoid arthritis trials.^15^, ^16^ The FDA further advised that data from children with polyarticular JIA or psoriasis, together with adult trials in rheumatoid arthritis, psoriatic arthritis, or psoriasis, could support extrapolation for juvenile psoriatic arthritis (JPsA) without a dedicated pharmacokinetic study. Consequently, extrapolation for juvenile spondyloarthritis (JSpA) has been applied on a drug‐specific basis, rather than a universal one.^17^

These regulatory advances culminated in the ICH finalizing the E11A guideline on pediatric extrapolation in December 2024.^18^ Extrapolation of adult efficacy data from rheumatoid arthritis and psoriatic arthritis supported the FDA approvals of intravenous golimumab for polyarticular JIA and JPsA in 2020,^19^, ^20^ as well as the FDA approvals of ustekinumab and guselkumab for JPsA.^19^, ^21^ Collectively, these developments reflect a maturation of pediatric trial design and regulatory science, enabling more efficient and ethically sound evaluation of novel therapies. Notably, this extrapolation framework was also applied in pSLE, when belimumab received FDA approval in 2019 based on extrapolation of efficacy from adult lupus trials, supported by pediatric pharmacokinetic, safety, and pharmacodynamic data.^22^

## Development of pediatric outcome and response measures for use in trials

### JIA

Over the past five decades, pediatric rheumatology has also benefited from the development of standardized pediatric outcome and response measures, which have made clinical trials more feasible and robust (Table 2). In the late 1990s, ACR pediatric JIA response criteria were first introduced (eg, JIA Pediatric 30, with additional thresholds such as Pediatric 50, 70, 90, and 100) to quantify graded improvement across six core variables in clinical trials.^13^ Building on this framework, Wallace and colleagues established consensus criteria for clinical inactive disease and remission in JIA in 2004,^23^ providing the first uniform definition of remission for oligoarticular, polyarticular, and systemic JIA. The Juvenile Arthritis Disease Activity Score (JADAS)^24^ was developed in 2009 as a continuous composite index that includes four core measures: physician disease activity assessment, parent/caregiver global assessment, active joint count, and acute‐phase reactants. Subsequent work defined cutoffs for states of inactive, minimal, moderate, and high disease activity.^25^ Additionally, the systemic JADAS was developed and validated for use in systemic JIA by adding a fifth core measure aimed at quantifying the activity of systemic features.^26^ Likewise, pediatric measures were developed to quantify disease activity in JSpA (the Juvenile Spondyloarthritis Disease Activity Index).^27^ Objective imaging measures, such as the Spondyloarthritis Research Consortium of Canada (SPARCC) magnetic resonance imaging inflammation and structural scoring systems, were also adapted for pediatric sacroiliac assessment to quantify inflammatory and structural lesions in juvenile SpA.^28^, ^29^ Additionally, standardized flare criteria for JIA^30^ and JSpA^31^ were developed.

**Table 2:** Pediatric‐specific trial response measures and outcomes by disease*

Disease	Core response measures	Disease activity/severity indices	Inactive disease/remission	Flare/damage/other key outcomes
JIA	Pediatric ACR response criteria (JIA ACR 30/50/70/90/100)^13^	JADAS (10/27/71)^24^	Wallace criteria for inactive disease^23^	JIA flare criteria^30^
	PRINTO provisional response criteria^25^	cJADAS^48^		JADI^49^
	Axial JSpA response criteria^47^	sJADAS^26^		SPARCC MRI inflammation and structural scores (for JSpA)^28^, ^29^
		JSpADA index^27^		
cSLE	PRINTO/ACR provisional criteria for response to therapy^35^, ^36^	pSLEDAI^32^	Consensus definitions for inactive disease and clinical remission^37^	Pediatric global flare criteria^38^
		ECLAM^33^		SLICC/ACR Damage Index (adapted for pediatrics)^50^
		SLAM^34^		
JDM	PRINTO/ACR/EULAR response criteria for JDM^41^	CMAS^40^	Definitions of inactive disease based on PRINTO criteria^51^	MDI^52^
		MMT‐8^42^		The Cutaneous Assessment Tool^53^
		DAS^39^		
jSSc	PRINTO provisional response criteria (under development/validation)	MRSS (pediatric use)^43^		
		J4S^54^		

* ACR, American College of Rheumatology; cJADAS, clinical JADAS; CMAS, Childhood Myositis Assessment Scale; cSLE, childhood systemic lupus erythematosus; DAS, Disease Activity Score; ECLAM, European Consensus Lupus Activity Measure; J4S, Juvenile Systemic Sclerosis Severity Score; JADAS, Juvenile Arthritis Disease Activity Score; JADI, Juvenile Arthritis Damage Index; JDM, juvenile dermatomyositis; JIA, juvenile idiopathic arthritis; JSpA, juvenile spondyloarthritis; JSpADA, JSpA Disease Activity; jSSc, juvenile systemic sclerosis; MDI, Myositis Damage Index; MMT‐8, Manual Muscle Testing in 8 muscles; MRI, magnetic resonance imaging; MRSS, Modified Rodnan skin thickness score; PRINTO, Paediatric Rheumatology International Trials Organisation; pSLEDAI, Pediatric Systemic Lupus Erythematosus Disease Activity Index; sJADAS, systemic JIA adaptation JADAS; SLAM, Systemic Lupus Activity Measure; SLICC, Systemic Lupus International Collaborating Clinics; SPARCC, Spondyloarthritis Research Consortium of Canada.

### Childhood‐onset SLE

For childhood SLE (cSLE), adaptations of adult disease indices, including the Systemic Lupus Erythematosus Disease Activity Index,^32^ European Consensus Lupus Activity Measure,^33^ and Systemic Lupus Activity Measure,^34^ have been applied and evaluated in childhood cohorts to assess disease activity and correlate with clinical outcomes. In 2009, the PRINTO/ACR provisional criteria for response to therapy in cSLE were developed.^35^ Building on this work, pediatric‐specific definitions of clinically meaningful improvement were developed and validated,^36^ along with thresholds for minor, moderate, and major clinical responses. Consensus definitions for inactive disease and clinical remission in cSLE were also established.^37^ Like JIA and JSpA, preliminary criteria for global disease flares in cSLE have been proposed for use in clinical trials.^38^

### Juvenile dermatomyositis

In juvenile dermatomyositis (JDM), a set of disease‐specific outcome measures has been developed and validated to facilitate clinical trials in this relatively understudied condition. Core assessments of disease activity include the Disease Activity Score (2003)^39^ and the Childhood Myositis Assessment Scale (1999),^40^ which provide complementary evaluations of muscle strength and functional capacity. Standardized response to therapy is assessed using the PRINTO/ACR/EULAR response criteria (2008),^41^ enabling harmonized outcome evaluation across trials. The Manual Muscle Testing in 8 muscles (2010)^42^ offers a practical and widely adopted measure of muscle strength. Together, these instruments have substantially accelerated therapeutic development in JDM by improving the sensitivity, comparability, and interpretability of clinical trial outcomes.

### Juvenile systemic sclerosis

In juvenile systemic sclerosis (jSSc), outcome assessment in clinical trials has focused on a combination of skin, organ‐specific, and global severity measures. The modified Rodnan skin thickness score (MRSS), adapted for pediatric use in 2010,^43^ remains a central measure of skin involvement, whereas overall disease severity is captured using the Juvenile Systemic Sclerosis Severity Score (2017).^9^ PRINTO has led efforts to develop and validate provisional response criteria for jSSc, though these measures remain under active refinement. At present, there are no universally accepted definitions of inactive disease or remission in jSSc.

### Patient‐reported outcomes

Additionally, cross‐cutting patient‐reported outcomes have assumed an increasingly prominent role in pediatric rheumatology trials.^44^ Over the past decade, the PROMIS pediatric measures, which are part of the NIH's Patient‐Reported Outcomes Measurement Information System, have been integrated into pediatric rheumatology trials to directly capture health‐related quality of life, pain interference, fatigue, and other domains from children and adolescents.^31^ Additionally, physical function is frequently evaluated using the Childhood Health Assessment Questionnaire. Lastly, recognizing the importance of treatment‐related harm, the pediatric Glucocorticoid Toxicity Index was developed in the early 2020s to systematically quantify glucocorticoid‐associated morbidity in children.^45^

## Conclusions

Over the past 50 years, the design, conduct, and impact of clinical trials in pediatric rheumatology have undergone a profound transformation, catalyzed by the establishment of collaborative research networks and registries that have enabled enrollment at scale and enhanced methodologic rigor. Coupled with strengthened regulatory incentives and internationally coordinated consortia, these advances have substantially improved the clinical trial landscape by accelerating therapeutic innovation while increasing the quality, relevance, and generalizability of evidence generated for children with rheumatic diseases. The pace of progress is unlikely to slow. Future advances are expected to include broader adoption of platform and adaptive trial designs, pragmatic and registry‐embedded trials, and Bayesian approaches tailored to small and heterogeneous populations. Advances need to also address barriers to trial enrollment from the perspectives of the patient/family and the clinical team. Continued efforts will focus on refining pediatric‐relevant outcome measures by developing harmonized core outcome sets, expanding patient‐reported outcomes, and integrating biomarker‐driven endpoints. In parallel, advancements in translational science, including biomarker discovery, precision medicine strategies, and preparedness for gene‐based therapies, will further shape the next era of pediatric rheumatology clinical research.

## AUTHOR CONTRIBUTIONS

All authors contributed to at least one of the following manuscript preparation roles: conceptualization AND/OR methodology, software, investigation, formal analysis, data curation, visualization, and validation AND drafting or reviewing/editing the final draft. As corresponding author, Dr Weiss confirms that all authors have provided the final approval of the version to be published and takes responsibility for the affirmations regarding article submission (eg, not under consideration by another journal), the integrity of the data presented, and the statements regarding compliance with institutional review board/Declaration of Helsinki requirements.

## Supporting information


**Disclosure form**.
